# Anterior cruciate ligament rupture during a cognitively demanding change-of-direction task in football: biomechanical insights from a laboratory injury

**DOI:** 10.1186/s12891-026-10338-6

**Published:** 2026-08-07

**Authors:** Dominic Gehring, Clara Ebner, Stefano Di Paolo, Francesco Della Villa, Urs Granacher

**Affiliations:** 1 Department of Sport and Sport Science, University of Freiburg, Sandfangweg 4, Freiburg, 79194 Germany; 2 Education and Research Department, Isokinetic Medical Group, FIFA Medical Centre of Excellence, Bologna, Italy

**Keywords:** Anterior cruciate ligament injury, Biomechanics, Neurocognitive demands, Injury mechanism

## Abstract

**Background:**

In laboratory-based injury risk assessments, there is always a small and not entirely avoidable risk of injury to participants. This case report describes an anterior cruciate ligament (ACL) re-rupture of a female participant during the approach run of a stimulus-response change-of-direction (COD) task.

**Methods:**

Marker-based kinematic data from the injury trial were compared with 14 successful trials of the same participant. These trials included unanticipated CODs in response to an opponent’s kicking action with varying cognitive demands, including one trial under identical high-demand conditions involving a feint. The participant approached at a speed of 4.08 m/s and the feint was initiated 475 ms before the injury step.

**Results:**

The injury trial differed from the non-injury trials in terms of a markedly prolonged flight phase preceding initial contact in combination with deviations in lower-limb and trunk kinematics during the flight phase and the step before. These deviations exceeded the participant’s typical movement range observed during the non-injury trials and included temporally pre-shifted flexion-extension patterns at the knee and hip, a more extended trunk at initial contact, an altered pelvic drop, and reduced ankle inversion. During the injury stance phase, pronounced hip adduction, knee abduction and ankle eversion were observed.

**Conclusions:**

The findings substantiate previously theorized mechanisms of non-contact ACL injury and show in a within-subject comparison that critical proximal and distal kinematic deviations at the trunk and lower limbs arise already during the preparatory phase. The occurrence after an opponent’s feint further suggests and highlights the importance of a neurocognitive contribution for ACL injuries.

**Trial registration:**

The study protocol was preregistered on Open Science Framework (10.17605/OSF.IO/4Z5R8).

**Supplementary Information:**

The online version contains supplementary material available at 10.1186/s12891-026-10338-6.

## Introduction

Anterior cruciate ligament (ACL) injuries represent a significant concern in team sports due to high incidence rates and long-term consequences for athletes [1, 2]. Understanding the underlying injury mechanisms is crucial for developing effective prevention strategies [3, 4]. A variety of methodological approaches have been employed to investigate these mechanisms, including interviews with injured athletes, biomechanical modeling and simulation, biomechanical laboratory studies imitating injury-related movements, and the analysis of injury occurrences through video recordings [5].

Research on ACL injury mechanisms in football revealed that ACL injuries mainly occur during horizontal movements like change-of-direction (COD) maneuvers, particularly in defensive pressing situations, often without direct opponent contact [6–9]. In addition, video analyses across various sports identified key biomechanical ACL risk factors such as an increased knee abduction angle, excessive hip adduction, and lateral trunk flexion [9–11].

Besides mere biomechanical ACL risk factors, the role of cognition during the injury incidence has received more attention in research over the past years. Non-contact football ACL injuries frequently occur during defensive maneuvers in high-pressure and time-restricted scenarios which require quick cognitive processing, decision making and finally reactive motor responses [12–14]. Moreover, injuries that occur during pressing actions frequently involve deceiving opponent actions such as feints happening roughly 250 ms before the initial ground contact [8, 15, 16]. The increased cognitive demands in these scenarios, which are caused by unpredictable and complex environmental conditions, may contribute to what Gokeler et al. called a ‘neurocognitive error’, resulting in altered neuromuscular control, ultimately increasing the ACL injury risk [14]. Cognitive factors associated with such injury-incidence situations include impaired decision-making, divided attention, reduced attentional inhibition, and diminished inhibitory control [16, 17]. For example, when responding to an opponent’s feint, adequate motor-response inhibition is needed to suppress an initial response in favor of an appropriate movement decision. A failure in these inhibitory processes (neurocognitive error) may influence neuromuscular control, potentially leading to impaired and injury-prone movement patterns [16]. These insights are limited, as they do not provide direct empirical evidence of the biomechanical and neurocognitive mechanisms underlying ACL injuries, leaving important aspects of the injury mechanism open to debate.

To address this limitation, laboratory-based experimental setups have frequently been used to mimic high-risk but non-injury situations that require substantial knee joint stabilization [5]. Accordingly, experimental setups have been developed to investigate the role of cognition on ACL injury-related biomechanics. In these experimental paradigms, anticipated movement conditions are often contrasted to unanticipated conditions (e.g., reacting to light signals, video animations, or real opponents). Alternatively, dual-task situations are employed to examine the effects of a secondary cognitive task on a football specific motor action (e.g., catching or handling a ball) [18]. Evidence from meta-analyses has shown that both, unanticipated conditions and dual-tasking appear to influence ACL injury-related biomechanical parameters during COD task performance or landing movements [18, 19]. Yet, while these findings arise from experimental approaches that allow for controlled testing of distinct risk factors, they ethically require safety margins, ensuring that participants can execute the movement tasks without any inherent injury risk. A key criticism of this approach is the uncertainty regarding whether the selected tasks and applied loads are comparable to real-world conditions associated with ACL injuries [5].

In laboratory-based injury risk assessments, there is always a small and not entirely avoidable risk of injury to participants. Accidental injuries occurring during biomechanical studies provide a unique opportunity to analyze injury mechanisms in unprecedented detail. By comparing a coincidentally happening injury trial with previous non-injury trials from the same participant under comparable conditions, case reports enable direct insight into the biomechanical factors associated with the actual injury event. Due to the relatively high incidence of ankle sprains, this type of injury has frequently been documented in laboratory settings. In 2022, Lysdal and colleagues summarized 12 ankle sprains that occurred during laboratory experiments and another 12 from competition, providing detailed insights into ankle joint kinematics and, in some cases, kinetics [20]. In contrast, reports of actual ACL injuries occurring during biomechanical research are fortunately rare. To our knowledge, only one case report exists documenting an ACL injury during a biomechanical study in skiing [21], along with another analysis using video footage during competition from a javelin thrower [22]. Both cases illustrate how comparisons between injury and non-injury trials can provide detailed insights into the ACL injury mechanism, especially when detailed biomechanical data from the same person under comparable conditions are recorded.

Here, we present a detailed single-case analysis of an ACL injury captured within a biomechanical research setting involving a female participant. This case report provides an unintentional but unique opportunity to examine the biomechanical factors involved in an actual ACL injury event. Specifically, this research focuses on an ACL re-rupture that occurred during the approach run for a COD movement following a feint of an opponent. This case allows for a detailed analysis of both biomechanical factors and temporal coupling between stimulus and motor response at the time of injury. Findings from this study provide valuable insights into biomechanical characteristics under cognitively demanding, highly dynamic situations that are directly related to an ACL injury.

## Methods

### Study design

This case report is embedded in a larger within-subject, repeated-measures study design investigating the effects of cognition on whole-body biomechanics during football-specific COD maneuvers in female athletes with different levels of football expertise [23]. For completeness, those aspects of the experimental design, data acquisition and analysis that are directly relevant to the present case are described below. The study protocol was preregistered on the Open Science Framework (10.17605/OSF.IO/4Z5R8). Ethical approval was obtained from the local ethics committee (University of Freiburg, #24-1142-S2).

### Case report participant

The case report focuses on a single female athlete aged 21 years (body height: 1.70 m, mass: 66 kg) who suffered an ACL re-rupture at the right knee during the approach run of a COD maneuver. Six years prior to study participation, the participant received ACL reconstruction with a semitendinosus graft after an alpine skiing injury. Imaging conducted after the recent laboratory incident revealed an ACL re-rupture with an additional lesion of the medial meniscus, as well as a posterior tibial slope of 18°.

The participant was initially part of the low-expertise football group with one year of experience as a student enrolled in sport science in collegiate football courses. The participant was initially part of the low-expertise football group, which comprised individuals with no more than one year of football-specific experience. She had one year of experience through collegiate football courses as a student enrolled in sport science. No eligibility criterion regarding overall physical activity level was applied. In total, the participant reported a weekly training volume of seven to ten hours of unspecific training including track and field, football, and swimming. Before testing, the participant met the inclusion criteria of being free of lower limb injuries within the last three months, of not having sustained an ACL injury or undergone ACL surgery in the preceding two years. In addition, the athlete did not report any other signs of being ineligible for study participation according to the PAR-Q questionnaire [24]. The participant provided written informed consent that allowed us to specifically analyze the single data set.

### Experimental setup and injury trial

The experimental setup was designed to replicate defensive playing situations in football. Participants performed COD tasks following an approach run at a target speed of 4.0 ± 0.3 m/s, monitored via timing gates in four block-randomized conditions with progressively increasing cognitive demands in each of the following conditions:

1. an anticipated 90° COD movement.
2. an unanticipated 90° COD movement with two movement options, i.e., to the left or right, in response to a real opponent kicking a ball in either direction at a standardized timepoint during the approach run of the participant,
3. inclusion of an additional third unanticipated movement option requiring the participant to either cut in response to a ball pass or to suddenly stop when the opponent halted the ball, and
4. inclusion of a feint condition, in which the opponent initially indicated a pass to one direction before quickly switching the supporting leg and passing the ball to the opposite side at the same timepoint as in the first two conditions.

To avoid fatigue and ensure consistent execution, participants were given rest intervals of approximately 30 s between trials. During all conditions, the opponent aimed to kick the ball as the participant was approximately 1.5 m from the center of the force plate, corresponding to an estimated time between kicking the ball and initial foot contact of the COD step at around 375 ms. This time interval between stimulus presentation and initial contact aligns with current recommendations for unanticipated COD testing protocols, aiming to simulate realistic decision-making demands while ensuring a safety margin for adequate motor responses [18]. Further details regarding the experimental setup and the test conditions can be found in the original research article containing group-based data [23]. Before data collection, participants completed a standardized warm-up protocol and at least three familiarization trials per condition to ensure adequate preparation for high-intensity movements.

The ACL injury occurred in the condition with the highest cognitive demand, after the participant had completed 14 COD trials across conditions with the right leg without any observable movement errors or discomfort. The injury incidence happened during the approach run one step before the penultimate step, i.e., two steps prior to the intended COD with the right leg. Thus, the injury occurred during the deceleration phase in the attempt to change direction, shortly after the opponent initiated a feint.

Of note, due to block-randomization, the participant had completed exclusively unanticipated COD tasks with the right leg prior to the injury event, i.e., seven trials in condition 2, four trials in condition 3, and three trials in condition 4, including one trial with the opponent’s feint. Additional COD trials performed with the left leg were not considered for the present analysis.

## Data collection and processing

Three-dimensional kinematics of the participant were captured at 200 Hz with a 12-camera marker-based motion analysis system (Vicon Motion Systems Ltd., Oxford, UK). A customized marker set comprising 37 markers placed on the participant’s head, trunk, pelvis, legs, and shoes was used based on established protocols [25]. To track stimulus initiation, a three-marker cluster was attached to the ball, and the opponent was equipped with 18 markers placed on trunk, pelvis, limbs, and footwear. Joint angles and segment orientations were subsequently calculated. Details regarding marker post-processing, segment definitions, joint center estimation, and the procedure for calculating joint angles and segment orientations in the global coordinate system can be found in Ebner et al. (2025) [23].

Subsequent analyses were performed in MATLAB (R2022b, The MathWorks Inc.). Kinematic time series from the actual injury trial were descriptively compared with the participant’s preceding non-injury trials. This included both the pre-injury trial performed under the same feint condition and further 13 pre-injury trials involving COD movements not including a feint. The analysis focused on joint angles in all three planes of the knee, hip, and ankle, as well as the relative trunk-to-pelvis angle. Additionally, the global orientations of the head, trunk, pelvis, and foot segments were determined. Biomechanical analysis commenced as soon as the athlete entered the capture volume. Because the foot of the injured limb was initially located outside the capture volume, kinematic data for this segment were available from 250 ms prior to IC for the injured foot, whereas all other body segments were captured from 350 ms prior to IC. The time interval for data analysis thus ranged from the ground contact phase of the step preceding the injury with the left foot and lasted to the end of the stance phase of the step with the right foot during which the injury occurred. Because ground reaction force data were unavailable for the injury step, the beginning and end of each stance phase were identified kinematically based on the horizontal velocity of the heel and toe markers exceeding or dropping below 1 m/s, respectively. This velocity-based criterion was selected following visual inspection of the marker trajectories and foot position, as it was considered to best reflect ground contact during the rapid deceleration preceding the COD maneuver.

To address the temporal aspects of the injury mechanism, stimulus-response times were calculated as the time intervals between distinct stimuli and the participant’s IC of the injury step. The stimuli to be considered were (a) the opponent’s initiation of the feint, being defined as the first movement of the feint foot exceeding a velocity threshold of 0.5 m/s, (b) the subsequent initiation of the kicking movement, being defined by the kicking foot that exceeded the same velocity threshold, and (c) the initial ball movement. Data processing steps were applied consistently across injury and non-injury trials to ensure methodological comparability.

## Results

### Spatiotemporal stimulus characteristics

A comparison of spatiotemporal parameters across conditions reveals that the injury and the uninjured feint trial show comparable stimulus characteristics (Table 1). In the injury trial, the feint was initiated 475 ms prior to IC of the injury step, corresponding to two steps before the injury event. The ball kick was initiated by the opponent 165 ms before IC, and thus during the stance phase of the step preceding the injury. The actual movement of the ball occurred during the stance phase of the injury step, i.e., 105 ms after IC.

**Table 1:** Spatiotemporal characteristics of stimulus presentation and approach run

Trial Type	Injury Trial	Uninjured Feint Trial	Reference Trials (Mean ± SD, [Full Range])
Feint initiation relative to IC (s)	-0.475	-0.490	n/a
Kicking movement initiation relative to IC (s)	-0.165	-0.145	-0.363 ± 0.027 [-0.320, -0.405]
Ball movement relative to IC (s)	0.105	0.135	-0.083 ± 0.042 [-0.035, -0.170]
Flight phase duration preceding injury step (s)	0.115	0.080	0.075 ± 0.015 [0.050, 0.105]
Speed of the approach run (m/s)	4.08	4.08	4.02 ± 0.10 [3.92, 4.17]

*IC *initial contact

The non-injury reference trials differed remarkably from the two feint trials regarding timing of the stimulus. Compared to the injury trial, both the kicking movement initiation and the subsequent ball kick occurred, on average, 188 ms and 198 ms earlier (Table 1).

With a duration of 115 ms, the flight phase preceding IC was remarkably longer in the injury trial compared to all other trials (Table 1).

## Biomechanics before injury ground contact

**Fig. 1:**
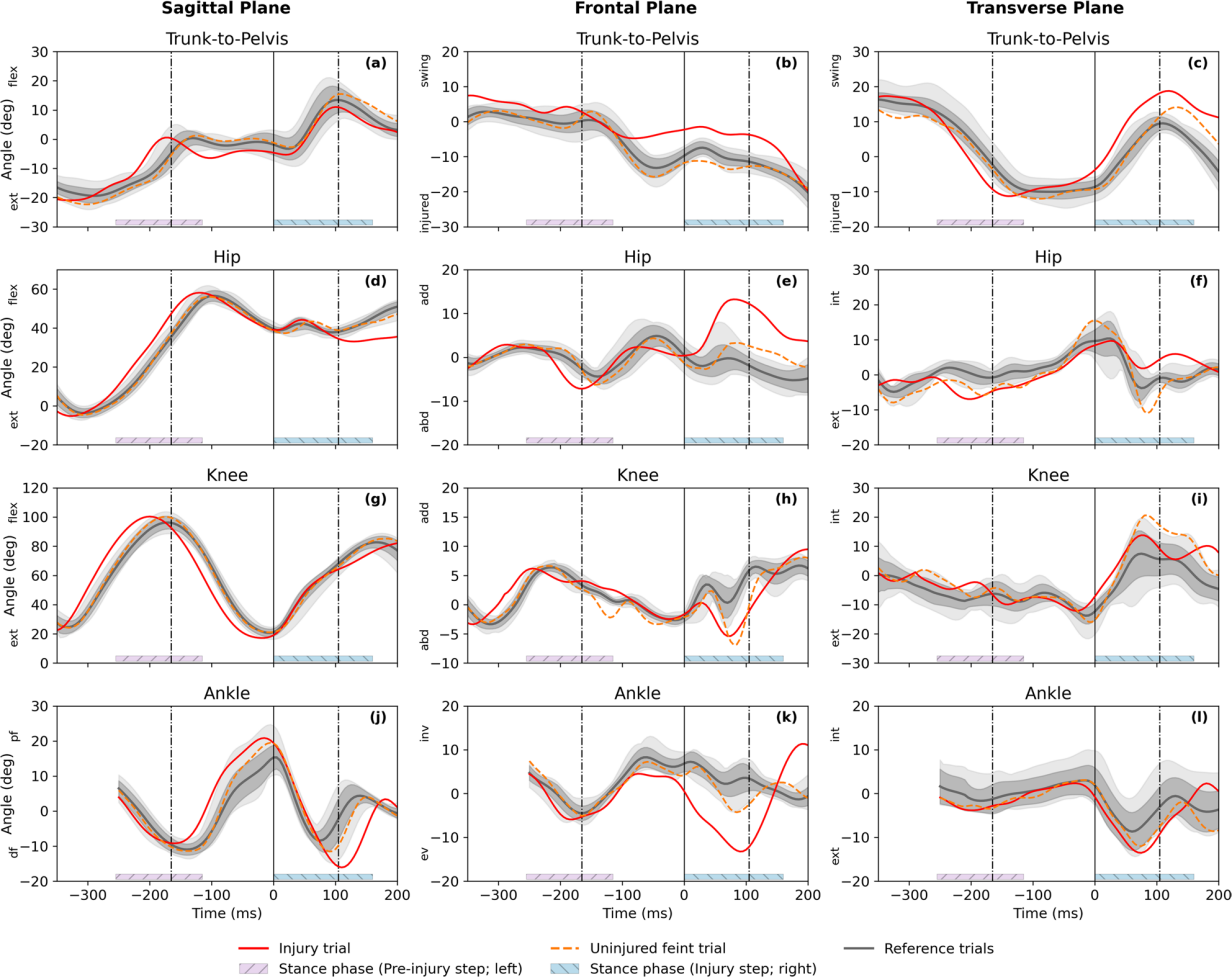
Joint angles of trunk-to-pelvis (panels **a**–**c**), hip (**d**–**f**), knee (**g**–**i**), and ankle (**j**–**l**) comparing the injury trial (red, solid line) with the uninjured feint trial (orange, dashed line) and 13 non-injured reference trials the mean (± SD in dark grey shaded and full range in light grey shaded) of the participant during the approach run to a change-of-direction. Time-series plots display joint angles from 350 ms before until 200 ms after initial contact of the injury step. Vertical dashed lines indicate two key stimuli of the opponent, i.e., kicking movement initiation (-165 ms) and ball movement (+105 ms), while the solid line highlights the initial contact. Transparent shaded regions mark the stance phases of the pre-injury step (left leg in light violet) and the injury step (right leg in light blue). Note that the illustration of stimulus timing and stance phases refer exclusively to the injury trial

**Fig. 2:**
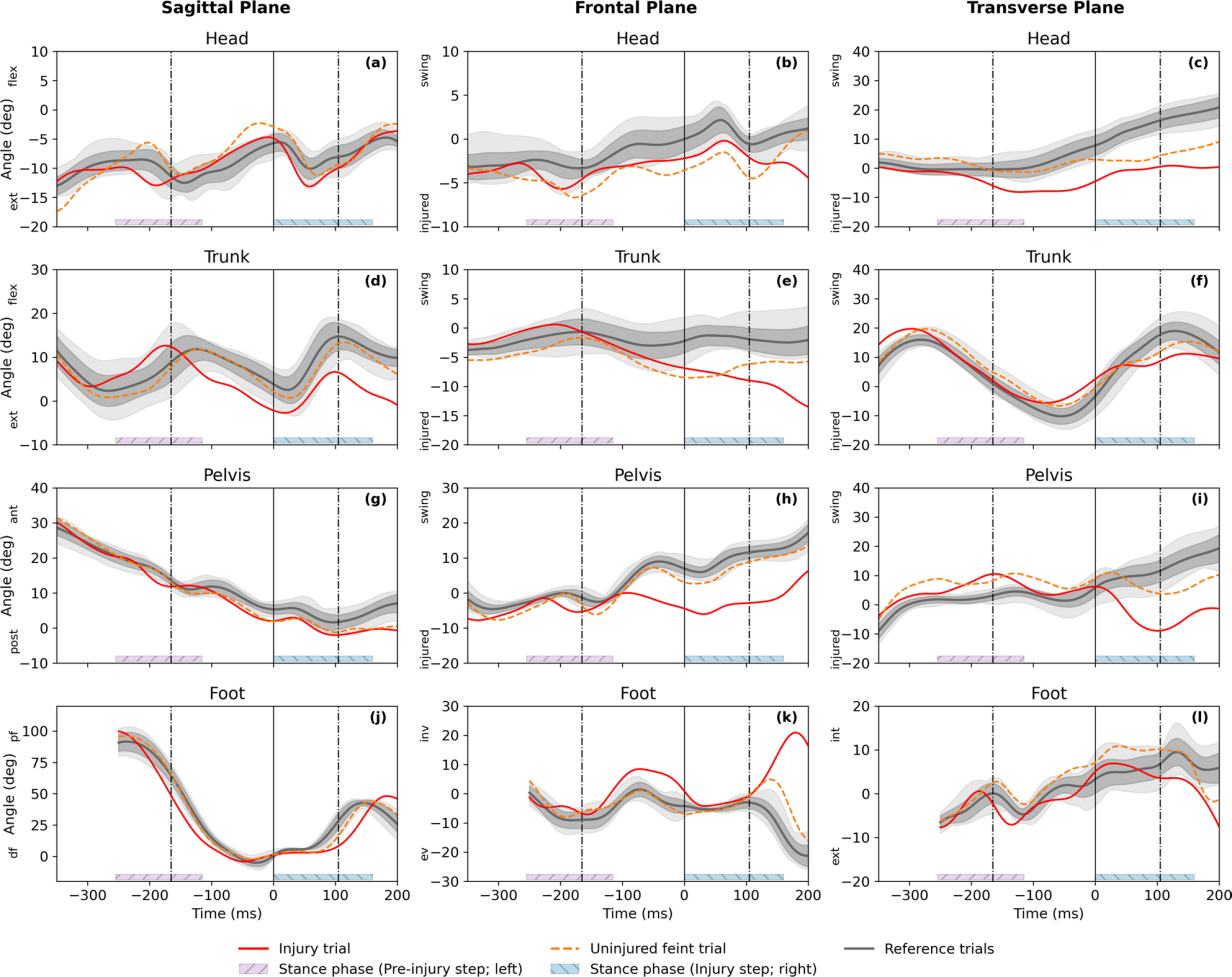
Global segment rotations of head (panels **a**–**c**), trunk (**d**–**f**), pelvis (**g**–**i**), and foot (**j**–**l**) comparing the injury trial (red, solid line) with the uninjured feint trial (orange, dashed line) and 13 non-injured reference trials the mean (± SD in dark grey shaded and full range in light grey shaded) of the participant during the approach run to a change-of-direction. Time-series plots display segment rotations from 350 ms before until 200 ms after initial contact of the injury step. Vertical dashed lines indicate two key stimuli of the opponent, i.e., kicking movement initiation (-165 ms) and ball movement (+ 105 ms), while the solid line highlights the initial contact. Transparent shaded regions mark the stance phases of the pre-injury step (left leg in light violet) and the injury step (right leg in light blue). Note that the illustration of stimulus timing and stance phases refer exclusively to the injury trial

Distinct temporal deviations in sagittal plane kinematics during the injury trial compared to all other trials were observed already at the beginning of the observation period, which also incorporated the step before the injury. Specifically, sagittal plane kinematic patterns at the knee and hip joints were temporally preceding by approximately 25 ms and 19 ms in the injury trial (Fig. 1d and g). This time shift was particularly pronounced at the knee joint and sustained up to IC of the injury step. The trunk was in a more erect position in space than in any of the other trials, beginning at the end of the pre-injury ground contact (Fig. 2d). At IC, this led to a trunk extension (backward lean) of -1.9° compared to an average forward lean of 3.9 ± 2.7° in the reference trials [full range − 1.0° to 8.2°].

In the frontal plane, substantial deviations were observed primarily at the level of pelvis and foot. Starting with the end of the pre-injury step’s stance phase and thus shortly after the kicking movement initiation, the pelvis dropped to the injured leg, in contrast to all other trials where a contralateral pelvic drop was observed (Fig. 2h). This pattern resulted in remarkable differences at IC of in average 11° comparing the injury with the reference trials. A corresponding alteration was observable in the intersegmental trunk-to-pelvis angle (Fig. 1b). In the same time interval, the ankle was more everted in the injury trial, as reflected by an almost neutral angle (1.6°) at IC, compared to the inverted positions observed in the reference trials (mean: 6.7 ± 1.7°; [4.6°, 9.5°]) (Fig. 1k).

Regarding transverse plane movements, the head was more rotated towards the injured leg, i.e., opposite to the new running direction, from early in the pre-injury step’s stance phase and thus around the time point of the kicking movement initiation (Fig. 2c).

**Fig. 3:**
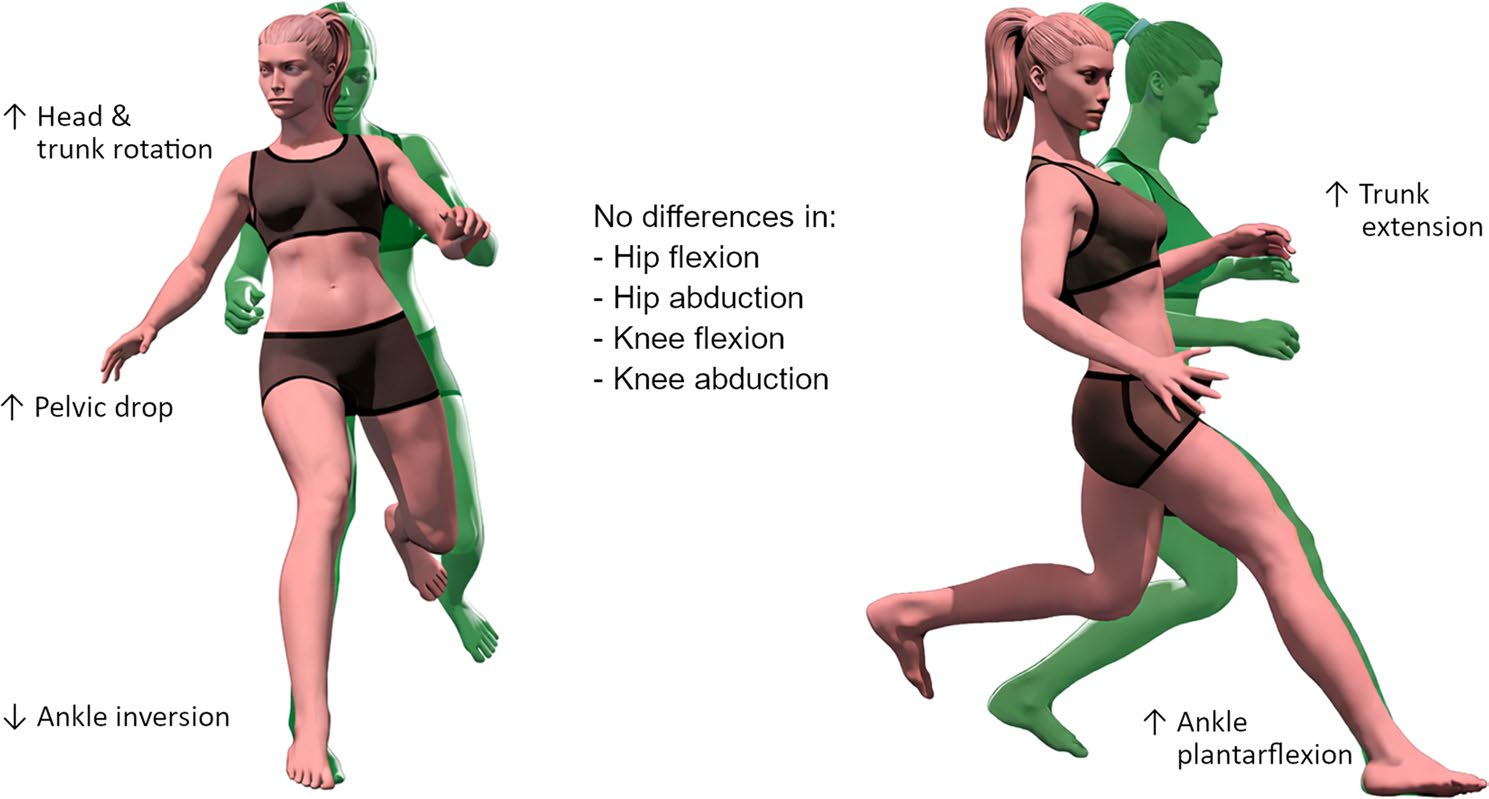
Graphical representation of the injury trial (red) and non-injury average trials (green) kinematics at initial contact in the frontal (left) and sagittal view (right). Descriptive differences within the figure refer to the biomechanics of the injury trial compared to the non-injury average trials

Figure 3 illustrates the key kinematic differences between the injury trial and the comparison trials at the time point of IC.

### Biomechanics during ground contact

In the injury trial, sagittal plane kinematic patterns began to deviate from those in the uninjured trials at approximately 90–100 ms after IC. Hip and knee remained more extended (Fig. 1d and g) and the ankle joint exhibited a more pronounced dorsiflexed angle (Fig. 1j) compared to the other trials. Furthermore, the foot remained prolonged in a flatfoot position on the ground, i.e., heel lift was delayed (Fig. 2j) and the trunk remained in a more extended position (Fig. 2d).

In the frontal plane, the hip exhibited pronounced adduction of up to 13.2° at 85 ms (Fig. 1e), accompanied by a sustained pelvic drop toward the injured leg (Fig. 2h). The ankle joint displayed a distinct eversion, reaching − 13.2° at 95 ms after IC in clear contrast to the reference trials (1.2 ± 3.0° [-4.3°, 4.6°] (Fig. 1k). The knee joint showed an abduction of up to -5.4° at 75 ms after IC. However, this did not clearly differ from the reference trials, and notably, the uninjured feint trial exhibited an even greater peak abduction of -6.9° (Fig. 1h).

Regarding transverse plane kinematics, a more pronounced trunk-to-pelvis rotation to the swing leg was observed, which corresponded to a clearly more pronounced rotation of the pelvis to the injured leg compared to the other trials.

The discrete whole-body kinematic values at IC for both the injury trial and the uninjured feint trial, along with the means and standard deviations for the reference trials, are provided in Supplement A.

## Discussion

This single-case report allowed for a detailed analysis of whole-body biomechanics and cognitive aspects of a non-contact ACL injury in a laboratory setting. We were able to contrast the injury trial against multiple non-injury trials of the same participant under comparable and highly standardized conditions. The main descriptive findings of this study are i) the injury displayed deviations in the participant’s movement pattern, that clearly exceeded the typical movement range especially at proximal and distal segments relative to the knee; (ii) the participant showed temporal and kinematic discrepancies already in the stance and flight phase prior the injury step; and (iii) the injury occurred in the condition with the highest cognitive demand including an opponent’s feint. This dataset therefore substantiates previously theorized mechanisms of non-contact ACL injury [11, 15, 16].

During the injury trial, the most pronounced deviations were observed not at the knee joint, e.g., knee abduction angle, but at proximal and distal segments. At IC, the athlete demonstrated atypical pelvic drop toward the injured leg, an increased ipsilateral trunk lean and rotation, a substantially larger ankle eversion angle, and even an unusual head rotation compared with her 13 preceding non‑injury trials. Rather than being attributable to the knee joint itself, the injury mechanism seemed more consistent with a whole‑body mal-coordination, where altered proximal control and altered distal positioning may have compromised the kinetic chain. This observation supports prior observational work covering ACL injury occurrences [8, 9], as well as laboratory experiments [25] and mechanistic theories [26] that emphasizes a multi‑segmental aetiology over isolated knee mechanics. Nevertheless, the absence of marked differences in knee abduction angle between the injury and non-injury trials does not preclude a contributory role of knee mechanics, which may have interacted with concurrent alterations in proximal and distal segment kinematics as part of a multifactorial injury mechanism. Accordingly, although this observation originates from a single case, it is consistent with previous video-based analyses of ACL injuries [8, 11] and further supports the rationale for incorporating whole-body control into ACL injury risk assessment and prevention strategies, alongside the evaluation of knee joint mechanics. From a mechanistic point of view, the ipsilateral pelvis drop combined with trunk lean, may have excessively increased knee abduction moments, by shifting the ground reaction force vector laterally relative to the knee joint [26]. However, this hypothesis cannot be verified, as no ground reaction forces were recorded during the injury step.

As these segmental deviations clearly exceeded the participant’s typical movement range, this data set furthermore supports the concept of an individualized “performance zone” where deviations from habitual patterns are associated with an increased injury risk [27]. In other words, in the injury trial, the participant’s execution variability [28] exceeded the behavioural flexibility, which can be defined as the ability to achieve the same task outcome using different movement execution solutions [29]. From this perspective, skilled movement emerges from an optimal balance between stability, enabling consistent task performance, and behavioural flexibility, allowing adaptation to changing task and environmental demands [29]. This might mirror real-world scenarios in football as players perform similar CODs repetitively during practice and competition, yet sustaining an injury in the one single event. Furthermore, it is possible that the participant’s relatively limited football-specific expertise reduced her behavioral flexibility, as skilled performers are thought to possess a broader repertoire of movement solutions that can be adapted to unexpected task demands [29]. Moreover, it might be speculated that habitual movement patterns and execution variability in individuals with a history of ACL injury differ from those of healthy controls even years after reconstruction [30, 31], as seen in this case.

Temporal kinematic discrepancies were observed noticeably before ground contact, suggesting a distortion of neuromuscular control very early in the preparatory phase of the injury trial. Specifically, already from approximately 350 ms before IC of the injury step, the hip and knee exhibited altered temporal flexion–extension patterns compared to non‑injury trials. This also relates to the elongated flight phase in the injury trial, suggesting a possible sensorimotor mismatch where anticipated ground contact timing and actual timing might become decoupled. This further highlights the requirements of appropriately preparing the IC, as for instance the kinematic state at IC largely predicts joint loading during the ground contact phase related to acute injuries like ACL-ruptures [32] or ankle sprains [33]. Consequently, both biomechanical analyses of injury mechanisms and ACL injury prevention strategies should consider the preparatory phase in addition to the kinematics at and after initial contact. Finally, the findings of the case align also well with situational patterns of non-contact ACL injuries during defensive pressing situations, where athletes failed to appropriately react to an opponent’s action during the pre‑contact phase [8, 16], making this particular time window specifically critical for injury prevention.

The injury occurred in the test condition with the highest cognitive and perceptual demand. Prior to the injury step, the athlete was confronted with an opponent’s feint initiated approximately 475 ms before IC, creating a narrow time window for stimulus processing, decision-making and motor execution. This temporal characteristic aligns with video data from non-contact ACL injury situations in football, showing a range from 40 ms to 560 ms between the opponent’s deceiving action and the IC of the injury step [16]. In these situations, motor‑response inhibition failures and attentional errors can be assumed [16, 34]. The present case thus supports this mechanism: the combination of high cognitive demand, temporal restrictions for response, and kinematic deviation in the preparatory phase before IC suggests that cognitive‑motor overload may have contributed to the failure, even in a safe and controlled laboratory environment. These observations align with previous evidence suggesting that evaluating movement quality under cognitively demanding, sport-specific conditions may provide additional insights beyond conventional testing of pre-planned movements [15, 16]. Together with the existing evidence, the present case report supports the inclusion of unanticipated COD tasks and decision-making demands in ACL injury risk assessment and prevention strategies.

The findings from this case report should be interpreted with caution, as it has several inherent limitations. First, as the analysis is related to a single participant with dedicated individual characteristics, such as a relatively low football-specific expertise and female sex, this limits generalizability of the results to athletes from other populations. Second, the participant had sustained an ACL injury and undergone reconstruction six years before the incident. Thus, the findings might solely reflect movement characteristics of athletes with previous ACL injury. Third, although we used a test setup that mimicked sport-specific demands therefore providing high ecological validity, the case happened in a controlled laboratory setting, which limits transferability to on-field situations. Fourth, since no kinetic parameters were recorded during the injury step, the actual impact of the striking kinematic parameters on knee joint loading cannot be quantified.

The presentation of this case report of a severe injury occurring in biomechanical research would be incomplete without ethical reflections. As we designed our study, we carefully elaborated and tested the experimental setup specifically focussing on how to provide a neurocognitively challenging yet safe environment. Despite the occurrence of the injury, the current protocol aligns with established testing standards. The applied approach speed and the stimulus-timing fall well within ranges of an available time to react from 216 to 1080 ms (median value 400 ms) and approach speeds from 3.0 to 5.5 m/s as reported in our recent meta-analysis [18]. Such neurocognitively enriched tests are requested and used in research but also in practical application like return-to-sports testing as they may provide unprecedented insights into the relation between motor-response inhibition and biomechanics [19, 35]. This would ultimately lead to preventive and rehabilitation strategies that train the athlete’s neuromuscular control more effectively. However, it should be noted that the use of neurocognitively enriched tests should carefully be considered, since they might place the athletes at greater risk of sustaining an ACL injury. Future studies employing such protocols should therefore aim to maximize neurocognitive demands while prioritizing participant safety. Accordingly, participant selection should carefully consider individual characteristics that may influence injury risk, and inclusion and exclusion criteria should be tailored to the specific demands and objectives of the experimental protocol. Furthermore, task constraints, including approach speed, cutting angle, and stimulus timing, should be selected based on established evidence from previously published experimental protocols [18] to ensure an appropriate balance between ecological validity and participant safety. Although it is impossible to completely eliminate the risk of injury during this type of testing, in accordance with ethical guidelines, the research team implemented all appropriate precautions to minimize risk. These measures were effective for all other 29 participants [23].

## Conclusions

This single-case report offers an unintentional opportunity to examine the biomechanical characteristics underlying a real ACL injury event during a cognitively complex COD task in a laboratory setting. The findings support previous evidence regarding biomechanical ACL injury mechanisms and highlight the contribution of proximal and distal segments, even during the movement preparatory phase. Furthermore, it can be hypothesized that a neurocognitive error may have contributed to the injury, as the IC of the injury step was immediately preceded by a deceptive feint of an opponent. Finally, we aim to raise awareness that biomechanical studies, even in a controlled and established setting, are not without risks for participants.

## Supplementary Information

Supplementary Material 1.

## Data Availability

The data is available from the corresponding author upon reasonable request.

## Abbreviations

ACL: Anterior cruciate ligament
COD: Change-of-direction
IC: Initial contact
