# Supplementary material for: Anterior cruciate ligament rupture during a cognitively demanding change-of-direction task in football: biomechanical insights from a laboratory injury
Source: BMC Musculoskelet Disord. 2026 Aug 7;27:660. doi: 10.1186/s12891-026-10338-6 (PMC13449388; doi:10.1186/s12891-026-10338-6)
Supplement: Supplementary file 1 [file 12891_2026_10338_MOESM1_ESM.docx]

**Supplementary material**

Supplement A Biomechanics at initial injury ground contact.

| **Trial Type** | **Injury Trial** | **Uninjured Feint Trial** | **Reference Trials  (Mean ± SD, [Full Range])** |
| --- | --- | --- | --- |
| **Intersegmental biomechanical parameters** | | | |
| Trunk-to-pelvis (sagittal)  (- = extension) [°] | -4.4 | -1.2 | -1.7 ± 3.5 [-7.4, 3.2] |
| Trunk-to-pelvis (frontal)  (- = to injured leg) [°] | -2.4 | -11.8 | -9.9 ± 2.0 [-12.8, -6.9] |
| Trunk-to-pelvis (transverse)  (- = to injured leg) [°] | -4.5 | -9.3 | -8.7 ± 2.0 [-12.7, -5.7] |
| Hip (sagittal)  (+ = flexion) [°] | 39.4 | 38.7 | 39.7 ± 1.7 [37.7, 43.3] |
| Hip (frontal)  (+ = adduction) [°] | 0.4 | -1.3 | 0.8 ± 2.0 [-3.6, 4.0] |
| Hip (transverse)  (+ = internal rotation) [°] | 8.1 | 15.5 | 9.6 ± 3.1 [4.3, 15.6] |
| Knee (sagittal)  (+ = flexion) [°] | 18.1 | 21.2 | 20.8 ± 2.6 [16.3, 24.6] |
| Knee (frontal)  (- = abduction) [°] | -1.9 | -2.1 | -2.3 ± 0.7 [-3.2, -0.9] |
| Knee (transverse)  (- = external rotation) [°] | -8.6 | -15.3 | -12.7 ± 2.7 [-20.2, -9.1] |
| Ankle (sagittal)  (+ = plantarflexion) [°] | 20.2 | 19.3 | 15.1 ± 3.6 [10.3, 24.4] |
| Ankle (frontal)  (+ = inversion) [°] | 1.6 | 4.2 | 6.7 ± 1.7 [4.6, 9.5] |
| Ankle (transverse)  (+ = internal rotation) [°] | 0.3 | 2.0 | 2.2 ± 3.9 [-1.9, 9.9] |
| **Global biomechanical parameters** | | | |
| Head (sagittal)  (- = extension) [°] | -4.7 | -2.9 | -5.8 ± 1.5 [-8.5, -3.8] |
| Head (frontal)  (- = to injured leg) [°] | -2.3 | -3.6 | -0.06 ± 1.5 [-2.1, 2.5] |
| Head (transverse)  (- = to injured leg) [°] | -5.0 | 3.0 | 7.6 ± 3.3 [2.5, 12.9] |
| Trunk (sagittal)  (- = extension) [°] | -1.9 | 2.1 | 3.9 ± 2.7 [-1.0, 8.2] |
| Trunk (frontal)  (- = to injured leg) [°] | -6.8 | -8.5 | -2.3 ± 2.0 [-6.4, 1.9] |
| Trunk (transverse)  (+ = to swing leg) [°] | 1.7 | -0.5 | -3.8 ± 3.3 [-10.2, 0.8] |
| Pelvis (sagittal)  (+ = anterior) [°] | 2.0 | 1.9 | 5.3 ± 1.4 [3.3, 7.6] |
| Pelvis (frontal)  (- = to injured leg) [°] | -4.2 | 3.1 | 7.1 ± 1.6 [4.4, 10.9] |
| Pelvis (transverse)  (+ = to swing leg) [°] | 6.2 | 9.1 | 5.5 ± 2.9 [-0.5, 11.4] |
| Foot (sagittal)  (+ = plantarflexion) [°] | 0.7 | 1.4 | -0.2 ± 2.1 [-2.3, 5.7] |
| Foot (frontal)  (+ = inversion) [°] | 2.2 | -6.9 | -4.3 ± 1.9 [-8.2, -1.0] |
| Foot (transverse)  (+ = internal rotation) [°] | 4.3 | 7.1 | 3.1 ± 3.1 [-2.7, 9.5] |
